# Measuring Party Identification in Public Opinion Surveys of Americans

**DOI:** 10.1093/poq/nfaf065

**Published:** 2026-02-23

**Authors:** Joshua J Dyck, Jack Santucci

**Affiliations:** Professor, Political Science Department, University of Massachusetts, Lowell, Lowell, MA, US; Professorial Lecturer, Department of Political Science, The George Washington University, Washington, DC, US; Lecturer, New York University, Washington, DC, US; and Senior Lecturer, Department of History, Philosophy, Political Science, and Economics, Western New England University, Springfield, MA, US

## Abstract

How should we measure “pure” or “true” independents? For years, the respective item required a respondent to volunteer that answer. Recent surveys have moved toward presenting it explicitly. Those that do produce estimates of *pure independents* that are much larger than in past surveys. We present evidence of this phenomenon across multiple surveys and ask: *Are self-administered surveys overcounting independents, or are traditional live-interviewer surveys undercounting independents?* We answer that question by comparing live-interview and self-administered samples from the 2012 and 2016 American National Election Studies, by undertaking tests to rule out mode effects (including an experiment), and by seeing which question wording correlates more strongly with measures of latent ideology, vote choice, and ratings of the parties. Our findings suggest that surveys that include an explicit response option, allowing Americans to self-identify easily as “(pure) independent,” offer a more precise measurement of the concept of party identification. This has implications for the study of independents, as well as for discussions about polarization and party-system dealignment.

## Introduction

*“Do you think of yourself as closer to the Republican Party or the Democratic Party?”*

Journalists and political scientists think about political independents in very different ways. In journalistic accounts, independents decide elections and are growing in number (Siders 2024). Political scientists generally see such accounts as sensationalism. Most independent identifiers “lean” toward either the Democratic or Republican Party (Keith et al. 1992), and independent identification for them is a way to hide their political commitments from friends (Klar and Krupnikov 2016). *True* or *pure* independents are actually scant in number and among the least-informed and -participatory groups in the electorate. What if the journalists are on to something? Might we have been measuring party identification poorly, pushing people into “leaner” bins who do not in fact lean? This paper presents evidence in favor of this “undercounting” claim. Said claim goes beyond the quality of journalism; partisanship may be the most important concept in the field of American political behavior (Campbell et al. 1960; Green, Palmquist, and Schickler 2002; Huddy, Mason, and Aaroe 2015).^1^

We use variation in survey practice to ask which way of measuring party identification is more accurate: putting the “(pure) independent” option before respondents or forcing them to offer it on their own.^2^ Assuming the centrality of party identification to people’s belief systems, this research question requires us to do two things. First, we need to show that larger shares of “(pure) independents” are not due to mode effects (i.e., using the internet). Second, we need to show that a measure based on an independent-inclusive item correlates more strongly with outcomes that are downstream of partisanship.

As we show below, surveys with the “(pure) independent” option systematically return much larger such populations than those without. To rule out mode effects, we point to the GSS (General Social Survey), which gives independents a “neither” option but uses live interviewers. Similarly, the CES (Cooperative Election Study) presents a “neither” option yet uses a self-administered (internet) format. We also summarize a survey experiment in which all respondents use the internet, but the treatment is the “neither” option. All of this casts doubt on mode effects.^3^ Then we use a 2012 switch in the way that the American National Election Study (ANES) asked about party ID to see which approach produces stronger correlates thereof. We find that the ANES item permitting independents to reject both parties is more strongly correlated with voting behavior, attitudes toward the parties, and a measure of latent ideology. These results suggest that it is a good idea to give respondents a “neither” or “independent” option at the second stage of standard party-ID questions, and it does not matter whether the survey is done online or with a live interviewer.

More broadly, as social scientists, we have been undercounting the share of “pure” independents for some time, pushing people into Democratic and Republican “leaner” categories. We mostly leave this measurement problem’s implications to the reader. However, we conclude with a call to pay more scholarly attention to independents, with the cautionary note that they are more numerous than commonly acknowledged in the literature.

## The Evolving Measurement and Interpretation of Party Identification

The rise of online/self-administered surveys in the last twenty years has created variation in how party identification is measured. For more than 60 years, political scientists used the following branching item or something similar to it. First, respondents are asked:

*Generally speaking, do you usually think of yourself as a Republican, a Democrat, an independent, or what?*

Those that answer *Republican* or *Democrat* are then asked about the strength of their identification:

*Would you call yourself a strong [Democrat/Republican] or not a very strong [Democrat/Republican]?*

Those who identify as *Independents* or *something else* are asked a different question:

*Do you think of yourself as closer to the Republican Party or the Democratic Party?*

Strong/Not Strong Democrats and Republicans form the outer bounds of the scale, points 1, 2, 6, and 7, respectively, in their answers to the first two questions. The third question fills out the middle of the scale. An independent or other-party identifier who says they *lean* toward the Democratic Party is a 3, and one who says they *lean* toward the Republican Party is a 5. Someone who volunteers the answer “no,” “neither,” “I’m an independent,” and so on, is classified as a 4, or what we normally call a “(pure) independent.”

The rise of online surveys created four possible states of the world for measuring party identification, and specifically for measuring “(pure) independents.” A survey can either be self-administered or have a live interviewer. Then, the branching question asked of independents can either put the option “no/neither/etc.” in front of respondents, or it can rely on the respondent to volunteer that answer. These states of the world are presented in table 1.

**Table 1.** Potential ways to measure “(pure) independent.”

	When measuring 7-point Party ID, the branching question for independents codes “(pure) independents” using a…	
Survey type	Forced-choice option (e.g., “neither” is a listed response option)	Volunteered response (e.g., respondents must volunteer a response other than those offered to them or skip the question)
Live interviewer	A (e.g., GSS)	B (e.g., most live-interviewer phone surveys, including the ANES)
Self-administered	C (e.g., ANES web sample since 2012, CES/CCES)	D (e.g., Pew American Trends Panel)

We call attention to cell D, when a survey is self-administered and the researcher wants to preserve the legacy surveys’ reliance on the volunteered response. This combination requires the researcher to give the respondent an alternative way to reject the binary choice of leaning. One way would be to provide the respondent with an open-ended option, while another would be to simply allow the respondent to skip the question. In practice, this has meant coding only skips as “pure independents,” effectively treating them as equivalent to the volunteered response in a live interview (Keeter 2019).

Most of what is written about independents is based on the approach in cell B. However, many researchers have begun using data based on the cell-C approach. Moreover, the field has not thought through the implications of variation in measurement of party identification, which is at the heart of the study of American political behavior (Campbell et al. 1960).

**Figure 1.**
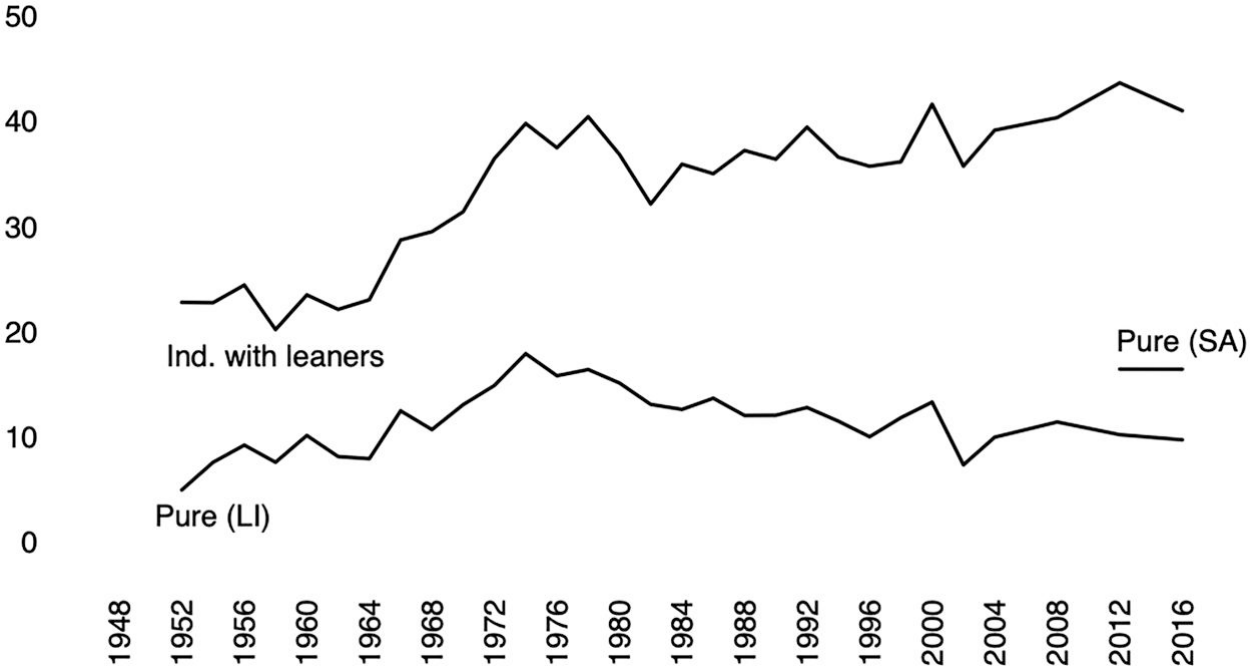
Percent of Americans who identify as Independent, 3-point and 7-point PID, 1952–2016 ANES (LI denotes a sample of respondents who were interviewed by a *live interviewer*, and SA denotes a sample who completed a *self-administered* survey. The graph distinguishes between Independents (with leaners included) and “(pure) independents.”

The distinction between “(pure) independents” and leaners is fundamental in public opinion research. In the 1970s, there was a growing trend in independent identification in the wake of the Vietnam War and Watergate scandal. Scholars posited a dealignment; Wattenberg (1998) argued that frustrated partisans began to shed their party identification as a rejection of both parties. Figure 1 shows the basic insight of the dealignment theorists. Americans increasingly began identifying as independent in the 1970s, and this trend has been relatively static over several decades, at least when we look only at the initial 3-point party identification response (ignoring the follow-up branch).

To be sure, political scientists have questioned much of dealignment theory. Keith et al. (1992) argued for a qualitative distinction between “leaning” and “pure” independents. Leaners, they argued, are better characterized as closet partisans because their voting behavior is not especially different from that of weak partisans. Independents who lean toward the Democratic Party act like Democrats, and those who lean toward the Republican Party act like Republicans. It is only those who are truly independent, the so-called “pure independents,” who act independently by voting for candidates of both parties and demonstrate unstable and inconsistent party loyalty rates. They also tend to act against stereotype by being the least informed and participatory group in American politics (Keith et al. 1992). This characterization, however, only holds for “(pure) independents,” not independent leaners. Leaners are partisans who are highly invested in keeping their partisanship a secret, and so they tend to be concerned with what others think *and* take steps to obfuscate their political identity (Klar and Krupnikov 2016).

Figure 1 begins to illustrate the measurement problem. Between 1952 and 2016, “(pure) independents” routinely have been estimated as 10–15 percent of the American public, as demonstrated in the lower time trend in figure 1.^4^ While the upper trend in the figure shows steep increase in independent identification starting in the 1970s (when we use the 3-point scale), *pure-independent identification does not follow this same pattern* (when we use the 7-point scale). Using the 3-point scale, we see that about 24 percent of Americans identified as independent between 1952 and 1968. However, after 1970, independent identification has averaged 36 percent of the public. But we do not see “(pure) independents” follow this pattern when we move to the 7-point scale. “(Pure) independents” were 9 percent of all Americans from 1952 to 1968, 13 percent of all Americans from 1970 to 2016, and 11 percent from 2000 to 2016.

Now consider the short trend beginning in 2012. This represents a move by the ANES to a two-pronged sampling approach with some respondents interacting with live interviewers and others filled out a self-administered web survey. This means that some respondents had their party identification measured using a cell-B approach (live-interview/volunteered-response) and others using a cell-C approach (self-administered/forced-choice).^5^ There is a clear difference in both years between “(pure) independents” in the self-administered/forced-choice (cell C) and live-interviewer/volunteered-response (cell B) versions of the ANES. “(Pure) independents” make up 16 percent of respondents in 2012 and 2016 in the self-administered/forced-choice sample (cell C) but just 10 percent in the live-interviewer/volunteered-response sample (cell B).

As a comparison point, the Cooperative Election Study (CES) codes party identification in the same way as the ANES online survey (i.e., table 1, cell C). Although the sampling strategy is slightly different,^6^ CES counts of “(pure) independents” look similar to those of the self-administered web version of the ANES, varying between 13 and 20 percent, depending on the year. This again suggests that self-administered and live-interviewer surveys are providing different measurements of party identification, particularly of “(pure) independents.”

The question remains: are differences in the estimates due to survey mode (self-administered vs. live-interview) or question wording (forced-choice vs. volunteered-response)? In the next section, we provide both observational and experimental evidence that this is a question-wording effect.

## Are Different Estimates of Pure Independents Due to Question Wording or Mode?

We take two approaches to determining whether the root cause of the measurement difference in “(pure) independents” in 2012 and 2016 is the nature of the questionnaire or the survey mode. First, we compare the ANES measurement of party ID to other data collections with and without live interviewers, including the CES, GSS, and Pew. Second, we present data from an experimental approach to measuring party ID using an original survey that we commissioned from YouGov. Overall, we find strong support for a question effect. Self-administered survey modes constrain how party ID may be asked, but it is the question, not the actual survey mode, that appears to be leading to differences in the measurement of party ID. When a forced-choice response option is offered that can be coded as “(pure) independent,” rather than when it is a volunteered response option, surveys are likely to report more independents. This is true of both online surveys and those administered by live interviewers (i.e., cells A and C). Our experimental evidence also suggests that it is difficult to replicate the conversational structure of branching party identification in web or self-administered surveys: offering the “neither” option leads to more respondents identifying as “(pure) independents,” and counting only skips almost entirely eliminates the category.

## Comparing the ANES to Other Surveys

As it turns out, there is a lot to be learned by looking at the variation in how survey organizations have approached measuring party identification. As survey organizations like Pew and the ANES have dealt with the move to online/web self-administered surveys, they faced the question of how to adjust their measurement strategy for party identification. Different organizations came to different conclusions. Additionally, the GSS stands as a bit of an outlier as a live interviewer survey. GSS respondents are provided a forced-choice response option of “neither,” allowing them to identify as “(pure) independents,” departing from the ANES and others who require a volunteered response. Returning to table 1, this places Pew’s American Trends Panel in cell D and the GSS in cell A, allowing for meaningful comparisons across surveys.

**Figure 2.**
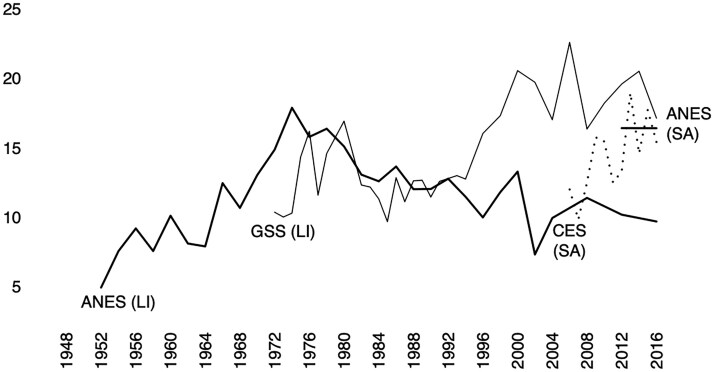
Pure Independents in the ANES, GSS, and CES, 1952–2016 (LI denotes a sample of respondents who were interviewed by a *live interviewer*, and SA denotes a sample who completed a *self-administered* survey).

In figure 2, we present a comparison of the ANES data with both the CES archive (back to 2006) and the GSS archive from 1970 to 2016. The ANES (LI) sample codes “(pure) independents” through a volunteered response, while ANES (SA), CES (SA), and GSS (LI) all provide an option for respondents to self-identify as pure independents by selecting “neither” in response to the branching question “Do you lean more toward the Democratic or Republican Party?” As you can see, little difference results from the approaches of the GSS (cell A) and ANES (cell B) through the 1970s and 1980s. However, starting in the 1990s, we see a clear divergence. Surveys that let respondents self-categorize explicitly as pure independents routinely have 50–100 percent more such people than surveys using a volunteered response.

The Pew Research Center’s data offer more evidence for a question-wording effect. Pew’s online survey is called the American Trends Panel (Pew Research Center 2019). In it, Pew does not provide respondents who say they are independent with an option to report “no,” “neither,” or “pure independent” in the subsequent branching question. Respondents are forced to choose Democrat, Republican, or to skip the question. The result has been a drastic decrease in the already small percentage of respondents classified as pure independent, from 7–10 percent in live interviewer surveys to typically less than 5 percent of the data in their self-administered web surveys. Using some recent Pew data,^7^ those who are classified as party ID refusals/skips make up only 271 of the 12,045 responses, 2.3 percent.^8^ Because of this choice, Pew no longer reports “(pure) independent” in their cross-tabulation reports in data that uses their web surveys since their measurement strategy (self-administered web survey, no forced-choice response option given for “(pure) independent”) does not allow for the meaningful classification of this category.

Examining the strategies of various survey research organizations provides evidence for a question effect, not a mode effect. To be sure, the need to change the party ID question approach stems from mode limitations for self-administered surveys, but the results of the various strategies to measure partisanship suggest that variations in question wording are meaningfully changing aggregate estimates of the size of the independent population.

## Experimental Evidence

Although evidence from the prior section strongly supports that differences in measurement of “(pure) independents” between surveys are being driven by question-wording and not mode effects, we use an experiment to bolster the case.^9^ Our experiment holds mode constant and varies the sets of response options offered on the independent follow-up question.

As part of a 2019 survey conducted by YouGov, 1,800 respondents answered an initial party identification question that asked, “Generally speaking, do you think of yourself as a …?” and were offered the following as response options: *Democrat*, *Republican*, *Independent*, *Other*, and *Not sure*. Another possible response was skipping this question altogether. Those selecting *Democrat* or *Republican* were then questioned on whether they call themselves strong or not-so-strong partisans. All other subjects (those selecting *Other*, *Not sure*, or skipping) were randomly assigned with equal probability (*p* = 1/3) to three different follow-up question formats. These formats varied by which combination of *Neither* and/or *Not sure* response options were included, alongside closeness to the major parties. Table 2 illustrates the question format treatments, referred to as Form A, Form B, and Form C.

**Table 2.** Partisanship question format text across experimental conditions.

Treatment	Follow-up question text	Response options
Form A	Do you think of yourself as closer to the Democratic or Republican Party?	The Democratic Party The Republican Party Neither Not sure
Form B	Do you think of yourself as closer to the Democratic or Republican Party?	The Democratic Party The Republican Party Not sure
Form C	Do you think of yourself as closer to the Democratic or Republican Party?	The Democratic Party The Republican Party

Form A shows two ways to avoid major-party identification, Form B offers one, and Form C does not list any non-major-party options (though in the latter, along with Forms A and B, respondents are coded as “(pure) independents” when they skip the question). A total of 605 respondents did not identify with either of the major parties on the initial partisanship question. Only these individuals were involved in the treatment assignment process, which resulted in 203 in Form A (includes “neither” and “not sure”), 198 in Form B (includes “not sure”), and 204 in Form C (excludes both). Those who answered as seeing themselves closer to the Democratic (Republican) Party were coded as Democrat (Republican) on the outcome variable for this experiment. Common partisanship coding practices dictate that these “leaners” are treated as partisans. Subjects answering with *Neither* or *Not sure*, as well as those showing up as missing after having answered the initial partisanship question (i.e., skipping the follow-up question), were coded as “(pure) independent.”

**Figure 3.**
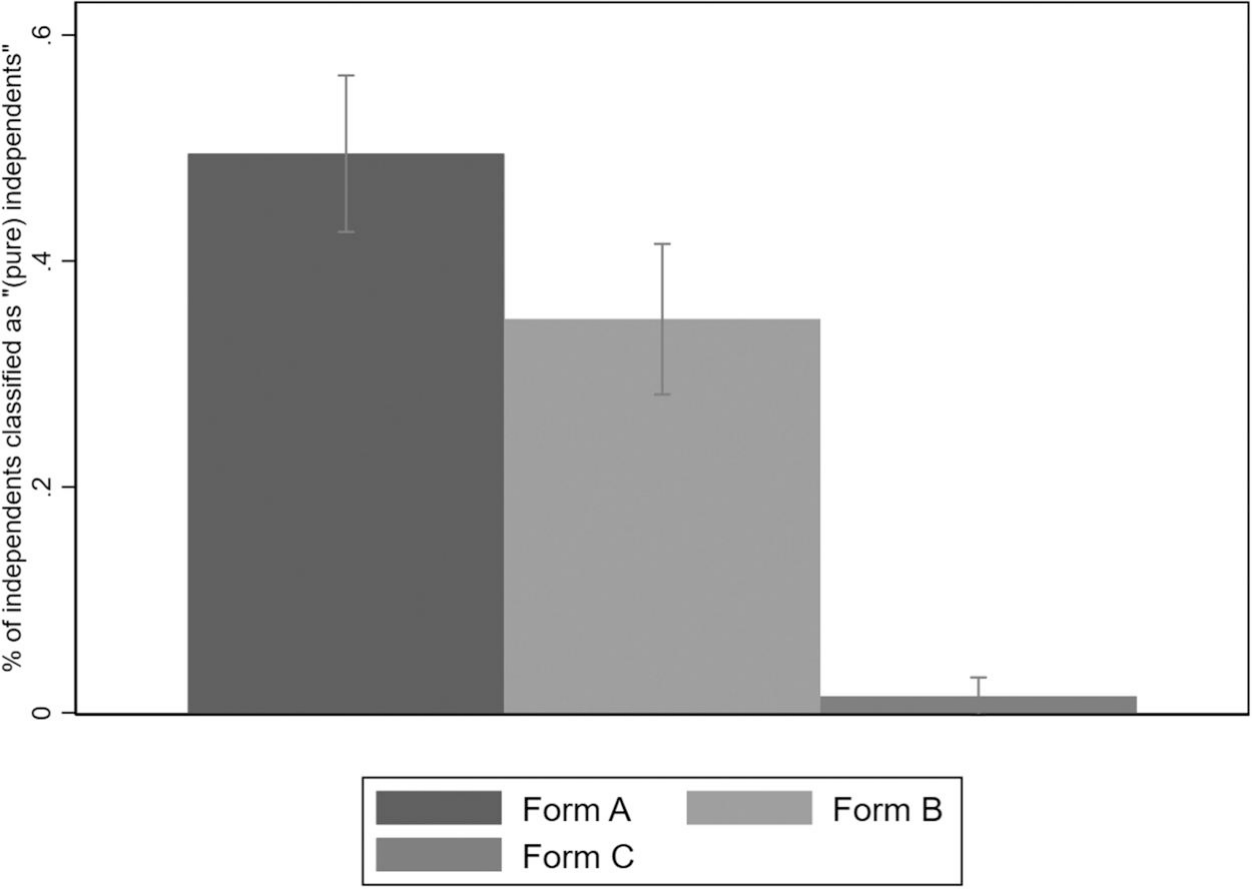
Question design affects how many independents are classified as “pure independents.” Data are from a 2019 survey conducted by YouGov. Percent of Independents who identify as “(pure) independents” by Question Format Treatments from table 2 (with 95 percent confidence intervals). N = 605 independents (Form A = 203, Form B = 198, Form C = 204).

In figure 3, we show the percentage of independent identifiers from the 3-point party ID scale who are then subsequently coded as *true* or *pure* independents, split out by treatment status. Bars include 95 percent confidence intervals. The results from this experiment offer evidence that question format matters tremendously for rates of independent identification on partisanship survey questions. In form A, when those initially identifying as independent are offered lean options and also both *Neither* and *Not sure* as responses, nearly half (49.3 percent) are classified as “(pure) independents.” That independent identification rate drops to 34.8 percent in Form B when *Neither* is no longer offered and plummets further to a mere 1.5 percent in Form C, when subjects can only escape major-party identification by skipping the follow-up question entirely.^10^

The key takeaway from this experiment is that the nature of the partisanship-question format affects the share of “(pure) independents” in surveys. When “neither” is provided as an option, more respondents ended up classified as “(pure) independents.” Furthermore, we clarified the insights of what happens in cell D from table 1; when respondents in a self-administered/forced-choice survey are given a binary option (Democrat/Republican) and only skips are coded as “(pure) independents,” the category is effectively eliminated.

As a final illustration of measurement sensitivity to question wording, we can calculate the partisanship distribution under different question formats by extrapolating partisan rates by treatment form to the entire pool of initial independent identifiers. Under Form A, a distribution of 47 percent Democratic, 36 percent Republican, and 17 percent independent results; for form B, it is 47 percent Democratic, 41 percent Republican, and 12 percent independent; and for form C, it is 51 percent Democratic, 48 percent Republican, and 1 percent independent.

Notably, offering *neither* provides a higher estimate of pure independents than has traditionally been found in live interviewer data that relies on the volunteered response. However, eliminating the “neither” response option and requiring a skip, as some organizations like the Pew Research Center do, provides a much smaller, almost negligible, estimate of “(pure) independents.” More research is needed here, but the self-administered version of the survey that produced an estimate of “(pure) independents” more in line with ANES live-interviewer, face-to-face estimates is one that omits the response option “neither” but offers “not sure” instead.

Overall, the experimental evidence supports what was gleaned by comparing the rates of independent identification across different survey collections (figure 2). The measurement of 7-point party ID, especially the measurement of “(pure) independent,” is highly sensitive to question wording. We conclude that it is very likely that the differences observed in the measurement of party identification between recent self-administered web surveys and those conducted with live interviewers (like the ANES) are the result of question wording. This is because, when we hold mode constant, “(pure) independent” identification is extremely sensitive to the instrument.

## External Validity—How Should We Measure Party Identification?

### Theoretical Perspectives

Our objective now turns to assessing the relative validity of a more inclusive measurement of “(pure) independents.” The case for measuring “(pure) independents” with a volunteered response has roots in two basic concepts in survey research design. One source of potential bias is the social desirability to appear independent, even if one is not particularly independent, given the widespread disdain Americans express toward parties (Hibbing and Theiss-Morse 2002; Klar and Krupnikov 2016). This point is made strongly by Baker and Renno (2019), who argue that offering *independent* as an option when respondents are first asked, “Generally speaking, do you usually think of yourself as a Republican, a Democrat, an independent, or what?” produces many false positives (partisans identifying as independents). Another potential form of response bias is that citizens who express any sort of ambivalence often pick the middle response (Tourangeau, Rips, and Rasinski 2000). The availability of no opinion/don’t know/neither are often the most desirable response options among respondents who (1) are trying to conceal their answers, (2) are attempting to expend the least amount of energy on a survey, and (3) have the lowest levels of educational attainment (Krosnick et al. 2001).

There are, however, some potential problems with the live-interviewer question approach. First, the reliance on a volunteered answer may introduce a different kind of bias; some respondents may believe that the question is simply a forced-choice question between Democrat and Republican. There also may be certain types of people that are less likely to challenge survey interviewers, thus creating a classification bias in who is identified as a “(pure) independent.” This could be due to group attachments, social identity, and/or socio-economic status. Furthermore, some of the concerns about social desirability bias that motivated the question approach for a live-interviewer survey are mitigated simply by moving from a live-interviewer survey to a self-administered survey. There is no interviewer to appease when a respondent is answering questions on a self-administered survey, and so those effects may be lessened or even completely attenuated.

Ultimately, the question of whether providing “neither” as a response option or relying on volunteered responses produces less bias is an empirical one. The task before us is to design a test or tests that allow for comparison of the measures of party identification. To do this, we take advantage of the natural experiment built into the 2012 and 2016 American National Election Study cross-sections.

Leveraging the variation in ANES in 2012 and 2016, we look at the simple relationships between party identification and: voting behavior, attitudes toward the parties, and latent ideology. The idea is that a more accurate measure of party ID leads to tighter correlations with attitudes toward “idea elements” downstream of party (Converse 1964). We also ask whether there are group-based and/or socioeconomic differences in the shares of “(pure) independents” in the two samples. The reason is that the live-interviewer/volunteered-response approach (again, cell B) may produce bias in who does and who does not identify as an independent based on who is willing to volunteer a response to the interviewer.^11^ Our empirical tests generally find that a more inclusive measure of party identification, like the ones used by the CES, GSS, and web portion of the ANES, are more strongly correlated with all three outcomes. Furthermore, we find some interesting demographic differences between the self-administered and live-interviewer sample of pure independents that lend further support to this perspective.

### Empirical Test 1—Party ID and Vote Choice

For several decades, it has been conventional wisdom in political behavior that independent leaners appear to be partisan because their behavior is partisan. That is, there is a stark contrast between independents who *lean* toward one of the two major parties and unattached, or pure independents. “(Pure) independents” have lower levels of general political knowledge, are less participatory, and leaners tend to be as supportive of their party nominees as weak partisans (Keith et al. 1992; Klar and Krupnikov 2016; Dyck and Pearson-Merkowitz 2023).

In table 3, we examine the difference in self-reported voting behavior in 2012 and 2016 by 7-point party ID using the ANES. In 2012, leaning Democrats and Republicans are more supportive of their party nominee in the self-administered/forced-choice sample than in the live-interviewer/volunteered-response sample. Additionally, a greater number of “(pure) independents” (16.6 percent vs. 5.1 percent) in the self-administered sample note that they voted for someone other than one of the two major-party candidates. In the 2016 data, leaning Republicans are more partisan in the self-administered sample, and there is no difference between leaning Democrats in the two samples.

**Table 3.** Cross-tabulations of Party ID x vote choice, 2012 and 2016 by survey mode.

2012 (f2f sample) Live interviewer (Pure Ind is a vol. response)				2012 (internet sample) Self-admin (Pure Ind is a forced-choice option)			
	Obama	Romney	Other		Obama	Romney	Other
Strong Dem	98.5	1.3	0.2	Strong Dem	97.8	1.2	1.0
Weak Dem	83.5	15.0	1.5	Weak Dem	82.9	14.9	2.1
Ind, leans Dem	85.2	12.5	2.3	Ind, leans Dem	90.1	5.6	4.2
Pure Ind	43.8	51.1	5.1	Pure Ind	46.3	37.1	16.6
Ind, leans Rep	12.9	83.8	3.2	Ind, leans Rep	5.0	87.9	7.0
Weak Rep	12.6	85.9	1.5	Weak Rep	11.7	85.4	2.9
Strong Rep	3.8	96.2	0.0	Strong Rep	2.2	97.2	0.6

2016 (f2f sample) Live interviewer (Pure Ind is a vol. response)				2016 (internet sample) Self-admin (Pure Ind is a forced-choice option)			
	Clinton	Trump	Other		Clinton	Trump	Other
Strong Dem	96.1	3.9	0.0	Strong Dem	96.5	2.5	1.0
Weak Dem	81.3	10.7	8.0	Weak Dem	75.7	17.7	6.7
Ind, leans Dem	80.3	5.6	14.2	Ind, leans Dem	80.1	8.5	11.5
Pure Ind	38.3	42.0	19.7	Pure Ind	33.9	44.2	21.9
Ind, leans Rep	9.1	76.4	14.5	Ind, leans Rep	7.8	83.2	8.9
Weak Rep	9.4	80.1	10.5	Weak Rep	19.9	71.3	8.8
Strong Rep	2.0	94.2	3.8	Strong Rep	2.8	95.4	1.9

*Note*: Cell entries are cross-tabulation column percentages.

Overall, then, the results for independents point to the self-administered sample as providing a measure of party identification that more accurately captures its relationship to voting behavior. That is, there is a stronger relationship between party ID and vote choice, particularly among leaners, in the sample that allows respondents to choose “neither” and thus self-select into the “(pure) independent” category. Furthermore, “(pure) independents” look more like a group that is truly uncertain or undecided in the self-administered sample, with a higher proportion of these respondents casting ballots for alternative/third-party candidates.

We get a similar result from another validity check using data from our 2019 YouGov survey. That survey lets us look at vote choice in 2016. We report those results in Appendix table C2. Also in Appendix C (table C1) are the ANES correlations between party ID (from each sample) and the party feeling thermometers. Again, the self-administered version of party identification produces a tighter correlation.

### Empirical Test 2—Ideology and Party ID

Our next validity check involves regressing each version of ANES party identification (7-point) on latent/operational ideology. The ideology score is constructed from 13 policy items that, through factor analysis, acceptably reduce to two dimensions (Treier and Hillygus 2009; Lupton, Myers, and Thornton 2015). Then we compare these regressions to see which version of party ID produces a better fit with ideology. We have an a priori expectation that party ID and ideology are strongly related (Carsey and Layman 2006; Chen and Goren 2016; Dyck and Pearson-Merkowitz 2023) and hence expect the better measure to be more strongly correlated with ideology.

Table 4 presents our results.^12^ The estimation approach is simple OLS, but we have suppressed the intercept, since we are only interested in the relationship between the two dimensions of ideology and party identification. Any additional factors affecting partisanship, such as political fundamentals or “valence” (Macdonald and Rabinowitz 1998; Schofield, Miller, and Martin 2003), would constitute the intercept term and thus could contaminate our test.

**Table 4.** OLS models predicting 7-point Party ID, 2012 and 2016 ANES.

	LI 2012	SA 2012	LI 2016	SA 2016
Ideology (1^st^ dimension)	1.047 (0.083)	1.509 (0.056)	0.644 (0.108)	0.971 (0.062)
Ideology (2^nd^ dimension)	0.373 (0.074)	0.812 (0.046)	0.663 (0.103)	0.794 (0.059)
R^2^	0.074	0.192	0.050	0.098
Adjusted R^2^	0.073	0.191	0.048	0.098
N	2033	3857	1166	3081

*Note*: LI refers to the samples that were administered by live interviewers, and SA refers to samples that were self-administered. Unstandardized regression coefficients and standard errors (in parentheses) are reported. The p-values for all coefficient entries in the table were infinitesimal and smaller than *p *< .0001.

The test shows that ideology in both dimensions is more strongly associated with 7-point party identification in the self-administered samples in both 2012 and 2016. We see this both from the beta coefficients and overall fit statistics, which show a greater percentage of variance explained in self-administered/forced-choice than live-interviewer/volunteered-response models. These results again suggest that offering “neither” as a response option correctly classifies more “(pure) independents” and allows for a more precise measurement of 7-point party identification, one that shows a stronger overall relationship with latent ideology.

### Empirical Test 3—Differences by Group Identity and Education

Finally, we look for differences in the makeup of “(pure) independent” populations by examining differences in race, gender, and education. Our analysis here is admittedly somewhat exploratory. However, we include this analysis because we believe there may be systematic differences in the underlying populations of pure independents between the live-interviewer/volunteered-response and self-administered/forced-choice versions of the survey. From a validity standpoint, we would want the underlying subgroup differences to be relatively innocuous, as large differences in “(pure) independents” moving from cell B to C from table 1 may reveal that the subpopulations are fundamentally different (and therefore that one should be preferred to the other). Additionally, probing by education allows us to see if there is a significant problem related to uncertainty and response bias. This is the central reason that “(pure) independent” has always been defined as a volunteered response; researchers worried that providing the category would misclassify partisans as “(pure) independents.”

In table 5, we present the differences in “(pure) independent” identification from the self-administered and live-interviewer samples of the 2012 ANES by gender, race, and education. What can we glean from this simple analysis? Notably, the differences are not monotonic in any of the categories. The largest percentage changes occur among respondents who identify as male, Black, and those with particularly high levels of education. That is, proportionally, there are more “(pure) independents” who are men, Black, and highly educated in the self-administered/forced-choice sample than in the live-interviewer/volunteered-response sample. There are a few interesting notes here. Perhaps the most relevant is the result for education. Recall that the literature on neutral response bias notes that *picking the middle answer because of its convenience* is most likely to occur among respondents with low levels of education. But here, we see independent identification as two to three times higher (the biggest difference) among those with the highest levels of education when *neither* is offered as an available forced-choice response option. This strongly cuts against the notion that offering respondents the option to clearly identify as a “(pure) independent” in the question (rather than requiring a volunteered response) would create a misclassification problem.

**Table 5.** Pure independents by gender, race, and education, 2012 ANES FTF and web surveys.

	Live interviewer (Pure Ind is a volunteered response)	Self-admin (Pure Ind is a forced-choice option)	Point change	% change
Gender				
% Female	11.1	16.0	4.9 points	44.1
% Male	9.2	16.9	7.6 points	82.6
Race				
% White	9.4	15.6	6.2 points	65.9
% Black	4.2	10.4	6.3 points	150.0
% Hispanic	14.6	22.5	7.9 points	54.1
Education				
No HS degree	18.3	27.2	8.9 points	48.6
HS degree	14.5	17.3	2.8 points	19.3
Some college	8.5	15.3	6.8 points	80.0
College degree	4.3	13.6	9.2 points	214.0
Graduate degree	5.6	12.6	7.0 points	125.0

*Note*: Cell entries in the first two columns are from cross-tabulations. The point change is column 2 minus column 1. Column 4 is the percentage change reflected as the value of column 3 divided by column 1.

Taking stock of all three findings, providing *neither* as a forced-choice response option appears to more accurately capture party identification. It provides a measure that is more strongly related to both vote choice and ideology, and there is some evidence that those who pick *neither* in the self-administered over the live-interviewer versions of the ANES are more highly educated.

## Conclusion

As costs have soared and response rates have declined for live-interviewer surveys, pollsters, academics, campaigns, and market researchers increasingly have turned to online formats as a cost-effective and accurate way to conduct survey research (Ansolabehere and Schaffner 2014). However, unbeknownst to many, the move to web surveys has changed the way that researchers measure party identification. We have shown that there is a fundamental difference in the population of “(pure) independents” when we shift to measuring them with a forced-choice question option as opposed to a volunteered response. The shift is important, as it leads to 50–100 percent more respondents in this category, and consequently, fewer leaning independents. Therefore, much of what we know about this population may be based on a biased undercount of the true independent population.

The fact that the population of “(pure) independents” is potentially much larger than previously thought is a finding that, in and of itself, deserves additional inquiry. Studies often disregard the importance of independents or swing voters, and a larger independent population might mean more persuadable voters in an election than previously thought. We also suspect our findings matter for polarization research. This is because the relationships between party identification and latent ideology, vote choice, and party affect, respectively, are all stronger when we use the forced-choice independent-inclusive measurement approach. Note that these measurement changes coincide with increasing attention to mass polarization in American politics. Studies that claim temporal increases in polarization may want to consider the underlying measurement strategy for party identification.

Given our findings, we end with a series of practical recommendations for researchers looking to properly measure party identification using a web survey:

- Our evidence suggests that the method of measuring party ID in the CES and web version of the ANES, which provides respondents the opportunity to identify as a *pure independent* in a forced-choice question format, produces the more empirically valid measure. It also produces higher estimated counts of independents in the population.
- Using the forced-choice option between Democrat and Republican and coding skips as “pure independent” essentially discards the concept, as well as that of independents generally. Surveys using this approach often categorize fewer than 5 percent of respondents as pure independents, and we have strong theoretical and empirical reasons to suggest that number is considerably higher.
- More research on independents is needed. We have an opportunity to now understand if independent identification has been misunderstood because of historical mismeasurement. This includes a need to more systematically study “(pure) independents,” a group that has been historically undercounted and is generally characterized as disaffected and not prone to political participation.
- Finally, from a measurement perspective, more needs to be done to understand just how sensitive party identification is to question wording effects.

## Data Availability

Replication data and documentation are available at https://doi.org/10.7910/DVN/3QNSES.

## Footnotes

1 To be sure, excellent work has measured partisanship cross-nationally. See, for instance: Bankert, Huddy and Rosema 2017; Huddy, Bankert, and Davies 2018; and Huddy, Davies, and Sandor 2020.

2 As we explain in section 2, the categories of 7-point party identification allow for classification of Americans from 1 (strong Democrat), 2 (Democrat, not strong), 3 (Independent, leans Democrat), 4 (Independent, no lean), 5 (Independent, leans Republican), 6 (Republican, not strong), 7 (strong Republican) from a series of branching questions. We will routinely refer to “(pure) independents” and those answering “no lean,” “just an independent,” or “neither” to the branching questions, those classified as a 4, interchangeably throughout the paper.

3 Pew Research Center uses online (self-administered) surveys but does not present a “neither” option (c.f. Keeter 2019).

4 Our analysis uses appropriate survey weights, consistent with the guidance from American National Election Studies, General Social Survey, and YouGov data used in analyses in this paper. All relevant information regarding weighting of data can be found in Appendix A. Handling of missing data is detailed in Appendix B2.

5 In 2012, the American National Election Study interviewed 5,914 respondents. Of these, 2,054 (35 percent) were from a traditional live-interviewer-administered face-to-face pooled-cluster sample, and the remaining 3,860 (65 percent) were from a self-administered survey collected online using a probability sample in partnership with Knowledge Networks. In 2016, the ANES replicated this method again, interviewing 4,270 respondents; 1,180 respondents were interviewed live, and 3,090 comprised a self-administered web sample.

6 The CES methodological approach uses convenience sampling, matching, and post-stratification to achieve a simulated representative sample.

7 We downloaded Wave #85 from the Pew American Trends Panel, from March 2021 (https://www.pewresearch.org/american-trends-panel-datasets/).

8 With the weight variable turned on, this is a highly underrepresented population in the overall dataset, and they are thought to account for 5.1 percent of the adult population.

9 In addition to differences in the format of the independent follow-up question, survey mode varies, perhaps meaningfully, as well. It is possible that the types of people who select into taking live or online interviews or political versus non-political surveys, or how they choose to respond to questions regardless of format, are correlated with independent party identification. CES data are collected using a convenience sampling methodology, and the GSS is a largely non-political survey.

10 These differences are statistically significant: (*p *= .003 for Form A vs. B, *p *< .001 for Form A vs. C).

11 For more on interviewer effects in survey research, see West and Blom (2017).

12 We use 13 common policy items between the 2012 and 2016 ANES and present the dimension-reduction analysis in Appendix table B1. The factor analysis uncovers two dimensions, which is typical for such exercises (see, e.g., Treier and Hillygus 2009). The content of the loadings does change between 2012 and 2016, consistent with issues that were uniquely salient in those presidential races. In 2012, there is a more typical split between economic and social policy issue dimensions, while the unique factors of the 2016 contest between Trump and Clinton elevated issues like immigration and moved racial politics to the second dimension.

## Appendix A. AAPOR-Required Disclosure Elements

**First data source:** American National Election Study, Cumulative Data File

### Data Collection Strategy

From the American National Election Studies: “In the ANES Time Series Cumulative Data File, the project staff have merged into a single file all cross-section cases and variables for select questions from the ANES Time Series studies conducted since 1948. Questions that have been asked in three or more Time Series studies are eligible for inclusion, with variables recoded as necessary for comparability across years.” More information and all *POQ* data disclosure items are part of the publicly available data archive here: https://electionstudies.org/data-center/anes-time-series-cumulative-data-file/.

### Research Sponsor and Conductor

The current PIs of the ANES are Nicholas Valentino and Shanto Iyengar. From 1952 to 1976, the ANES was funded through the University of Michigan, and since 1977, it has been an NSF-funded study. You can read more about the history here: https://electionstudies.org/about-us/history/.

### Measurement Tools & Instruments/Population Under Study/Methods Used to Generate and Recruit Sample/Methods and Modes of Data Collection/Dates of Data Collection/Weighting

From the ANES: “Over the years, the most common ANES study design has been a cross-section, equal probability, sample. These designs are typically ‘self-weighting’—i.e., the respondents do not need to be weighted to compensate for unequal probabilities of selection in order to restore the ‘representativeness’ of the sample. On several occasions, however, ANES has departed from this standard design. In some years, ANES ‘oversampled’ certain groups (African-Americans in 1964, for example). In other years, the Election Study combined a panel reinterview with a cross-section design (as in 1974, for example). It is important to understand that the Time Series Cumulative File is a file of pooled cross-section studies: any respondent for a particular study who is strictly ‘panel’ or ‘supplement’ has been deleted from the Time Series Cumulative File. For example, the sequence of studies 1972, 1974, and 1976, constitutes a panel, with cross-section respondents in 1972 and 1974 being reinterviewed in succeeding years. If a 1972 respondent moved out of the SRC sampling area, but was nevertheless reinterviewed, that respondent became a ‘panel only’ respondent, and the representativeness of the 1974 cross-section was maintained by selecting a new respondent from the residents at the sample address from which the ‘panel only’ respondent had moved. Such 1974 ‘panel only’ respondents are not included among the 1974 respondents that appear in the Time Series Cumulative Data File.

Because not all of the cross-section samples included in the Time Series Cumulative Data File are equal probability and thus self-weighting, all pooled cross-section descriptive analyses should be run using Variable 9, the weight variable. For most years, the value of that variable for all respondents is simply ‘1.0.’”

All data in the present paper were weighted using the conventions suggested herein.

Readers are directed to the Cumulative Data File codebook here: https://electionstudies.org/anes_timeseries_cdf_codebook_var_20220916/.

### Sample Sizes/Disposition or Response Rates/Details about Interviews and Interviewers

It should also be noted that sample sizes, response rates, and sample-specific details are available on each individual study’s page by referencing the “User Guide and Codebook” pdf. These files have subsections for each study year that detail the full questionnaires and methodological/study details.

**Measurement and Model Specification:** A complete replication packet, including variable selection, coding, and model specifications, is available here: https://doi.org/10.7910/DVN/3QNSES.

### A General Statement Acknowledging Limitations of the Design and Data Collection

All surveys are subject to potential bias related to both sampling and questionnaire design. We provide full transparency of all data collected, models estimated, and coding decisions made throughout the manuscript and in our replication materials, such that interested readers are easily able to follow and replicate our analysis to scrutinize them. In working with survey data, there are always potential limitations in generalizability that may arise from the ability of the data generation modeling process to accurately represent the underlying population of interest.

**Second data source:** General Social Survey

### Data Collection Strategy

The General Social Survey is a long-term repeated cross-sectional survey of social trends that has repeated questions from 1972 to 2018. Details about the survey, its genesis, and data collection strategies are available in the codebook on pages viii–x. See: https://gss.norc.org/content/dam/gss/get-documentation/pdf/codebook/GSS_Codebook_intro.pdf.

### Research Sponsor and Conductor

The General Social Survey is conducted by the National Opinion Research Council (NORC) at the University of Chicago. See more here: https://gss.norc.org/us/en/gss/get-documentation.html.

### Population Under Study

The population under study is American adults, aged 18+.

### Measurement Tools & Instruments/Methods & Modes of Data Collection/Methods Used to Recruit the Sample/Dates of Data Collection/Sample Sizes/Precision of the Results/Weighting/Data Processing/Interviewers and Interviewing/Dispositions and Response Rates/Sample Sizes

The GSS was completed every year from 1972 to 2018, and the codebook details variations in sample sizes, dispositions, questions, weighting, data collection, and so on, in a detailed section of the codebook, linked online and publicly available here: https://gss.norc.org/content/dam/gss/get-documentation/pdf/codebook/GSS_Codebook_AppendixA.pdf.

**Measurement and Model Specification:** A complete replication packet, including variable selection, coding, and model specifications, is available here: https://doi.org/10.7910/DVN/3QNSES.

### A General Statement Acknowledging Limitations of the Design and Data Collection

All surveys are subject to potential bias related to both sampling and questionnaire design. We provide full transparency of all data collected, models estimated, and coding decisions made throughout the manuscript and in our replication materials, such that interested readers are easily able to follow and replicate our analysis to scrutinize them. In working with survey data, there are always potential limitations in generalizability that may arise from the ability of the data generation modeling process to accurately represent the underlying population of interest.

**Third data source:** YouGov survey, July/August 2019

**Data Collection Strategy:** Survey of American adults

**Research Sponsor and Conductor:** UMass Lowell sponsored the survey and YouGov, Inc., conducted it.

**Measurement Tools/Instruments:** A complete replication packet, including variable selection, coding, and model specifications, is available here: https://doi.org/10.7910/DVN/3QNSES. We also refer readers to table 2 of the main text of the article for the logic of the experiment’s question wording.

**Population Under Study:** American adults, aged 18 and older

**Methods Used to Generate and Recruit the Sample:** Opt-in non-probability from YouGov’s online panel. YouGov interviewed 2,229 respondents who were then matched down to a sample of 1,800 to produce the final dataset. The respondents were matched to a sampling frame on gender, age, race, and education. The frame was constructed by stratified sampling from the full 2016 American Community Survey (ACS) one-year sample with selection within strata by weighted sampling with replacements (using the person weights on the public use file). The matched cases were weighted to the sampling frame using propensity scores. The matched cases and the frame were combined, and a logistic regression was estimated for inclusion in the frame. The propensity score function included age, gender, race/ethnicity, years of education, and region. The propensity scores were grouped into deciles of the estimated propensity score in the frame and post-stratified according to these deciles.

**Method(s) and Mode(s) of Data Collection:** Web

**Dates of Data Collection:** July 22, 2019–August 12, 2019

**Sample Sizes (by sampling frame if more than one frame was used) and (if applicable) Discussion of the Precision of the Results:** N = 1,800 American adults

**Whether and How the Data Were Weighted:** A post-stratification weight variable was calculated based on 2016 presidential vote choice, and a four-way stratification of gender, age (four categories), race (four categories), and education (four categories).

**How the Data Were Processed and Procedures to Ensure Data Quality:** YouGov data collection includes attention checks.

**Screening Criteria and Process:** Respondents were pre-screened for inclusion in the sample based on information in YouGov’s panel.

**Dispositions or Response or Participation Rates:** It is not appropriate to include response rates for non-probability samples, particularly for YouGov samples, since (1) the sampling system used is dynamic in nature and assigns users to open surveys once they click on a survey link, (2) YouGov’s approach is to model the underlying population of interest, rather than to sample it, and (3) the underlying AAPOR consensus is not to include response rates for non-probability online panels.

**Measurement and Model Specification:** A complete replication packet, including variable selection, coding, and model specifications, is available here: https://doi.org/10.7910/DVN/3QNSES.

**A General Statement Acknowledging Limitations of the Design and Data Collection:** All surveys are subject to known biases, including sampling and response bias. YouGov’s approach to modeling has performed well in external validity checks that mirror high response rate probability samples. However, there are limitations in generalizability that may arise from the ability of the data generation modeling process to accurately represent the underlying population of interest.

**Fourth data source:** Cumulative CES Common Content (Kuriwaki 2024)

**Data Collection Strategy:** The CES is an annual cross-sectional data collection effort that interviews over 50,000 Americans about elections. Kuriwaki (2024) has created a single file across all years (2006–2024) to track common content over time: Notably, the “dataset contains the respondents from the Common Content of the CCES (n = 701,955), combining all available Common Content datasets from 2006–2024. It includes select standardized variables including demographics, geography, vote choice, validated vote, representative approval, the economy, etc.”

https://dataverse.harvard.edu/dataset.xhtml? persistentId=doi%3A10.7910/DVN/II2DB6

### Research Sponsor and Conductor

Currently, the CES is hosted and sponsored by Jonathan M. Tisch College of Civic Life at Tufts University. https://tischcollege.tufts.edu/research-faculty/research-centers/cooperative-election-study/data-downloads-and-tools-scholars

### Measurement Tools/Instruments

The Cumulative CES file collects common items across 19 years of CES data. This is detailed in Kuriwaki’s (2024) codebook, specifically on pages 13–42.

### Population Under Study

American adults, aged 18 and over

### Measurement Tools & Instruments/Methods & Modes of Data Collection/Methods Used to Recruit the Sample/Dates of Data Collection/Sample Sizes/Precision of the Results//Data Processing/Sample Sizes

While it varies slightly from survey to survey, the CES largely recruits its respondents from YouGov and then uses a propensity score matching and deletion with post-stratification method to simulate a representative sample from an online convenience sample. For specific details on data collection dates and sampling, readers are referred to the methodological details of each individual survey year, available here: https://dataverse.harvard.edu/dataverse/cces.

### Whether and How the Data Were Weighted

Weighting details for the full cumulative file are detailed by Kuriwaki (2024) on pages 11–13 of the codebook.

### Dispositions or Response or Participation Rates

It is not appropriate to include response rates for non-probability samples, particularly for YouGov samples, since (1) the sampling system used is dynamic in nature and assigns users to open surveys once they click on a survey link, (2) YouGov’s approach is to model the underlying population of interest, rather than to sample it, and (3) the underlying AAPOR consensus is not to include response rates for non-probability online panels.

**Measurement and Model Specification:** A complete replication packet, including variable selection, coding, and model specifications, is available here: https://doi.org/10.7910/DVN/3QNSES.

**A General Statement Acknowledging Limitations of the Design and Data Collection:** All surveys are subject to known biases, including sampling and response bias. YouGov’s approach to modeling has performed well in external validity checks that mirror high response rate probability samples. However, there are limitations in generalizability that may arise from the ability of the data generation modeling process to accurately represent the underlying population of interest.

**Fifth data source:** American National Election Study, 2012 Time Series Study

**Data Collection Strategy:** The 2012 ANES was a dual mode survey conducted on attitudes about American elections, the 29^th^ cross-section in a wave conducted in the series.

**Research Sponsor and Conductor:** National Science Foundation, University of Michigan, Stanford University.

### Measurement Tools/Instruments

The Complete Codebook for the 2012 ANES is available here: https://electionstudies.org/wp-content/uploads/2012/02/anes_timeseries_2012_userguidecodebook.pdf.

### Population Under Study

American adults, 18+

### Methods Used to Generate and Recruit the Sample

Details about sample recruitment are available in the study’s User Guide and Codebook. For the face-to-face mode, address-based sampling was used with in-person recruitment and interviews. For the internet mode, it was primarily address-based sample and mail recruitment, followed by internet interviews. However, some RDD telephone sample and recruitment was also used, followed by internet interviews. More details here (pages 28–30): https://electionstudies.org/wp-content/uploads/2012/02/anes_timeseries_2012_userguidecodebook.pdf.

### Method(s) and Mode(s) of Data Collection

Dual-mode (in-person/internet sample). Details above in the codebook.

### Dates of Data Collection

Pre-election, face-to-face: Sept. 8–Nov. 5 (median October 13).

Pre-election, internet: Oct. 11–Nov. 6 (median Oct. 25).

Post-election, face-to-face: Nov. 7–Jan. 13, 2013 (median Nov. 26).

Post-election, internet: Nov. 29–Jan. 24, 2013 (median Dec. 9).

**Sample Sizes (by sampling frame if more than one frame was used)**

Pre-election: 5,914 (2,054 face-to-face and 3,860 online)

Post-election: 5,510 (1,929 face-to-face and 3,581online)

**Discussion of the Precision of the Results**

DEFF for the full sample is 1.8076

Pre-election sample MOE: +/-1.7 percent

Post-election sample MOE: +/-1.8 percent

### Whether and How the Data Were Weighted

Discussion of weighting is available in the codebook (linked above) on page 32. Analyses using these data were conducted using the appropriate weights.

### More Information about the ANES (Data Processing & Data Quality/Interviewers/Study Stimuli) Can be Found in the Codebook

https://electionstudies.org/wp-content/uploads/2012/02/anes_timeseries_2012_userguidecodebook.pdf

### Dispositions or Response or Participation Rates

In-person sample: 38 percent (RR1)

Online sample: 2 percent (RR1)

**Measurement and Model Specification:** A complete replication packet, including variable selection, coding, and model specifications, is available here: https://doi.org/10.7910/DVN/3QNSES.

### A General Statement Acknowledging Limitations of the Design and Data Collection

All surveys are subject to potential bias related to both sampling and questionnaire design. We provide full transparency of all data collected, models estimated, and coding decisions made throughout the manuscript and in our replication materials, such that interested readers are easily able to follow and replicate our analysis to scrutinize them. In working with survey data, there are always potential limitations in generalizability that may arise from the ability of the data generation modeling process to accurately represent the underlying population of interest.

## Appendix B. Measurement Details for Policy-Based Ideology

1. Variables used to construct policy-based ideology in two dimensions in 2012 and 2016:
  - VCF0809: Guaranteed Jobs and Income Scale
  - VCF0806: Government Health Insurance Scale
  - VCF0830: Aid to Blacks Scale
  - VCF0823: Better off if U.S. Unconcerned with Rest of World
  - VCF9230: Fed government should encourage/discourage the use of foreign workers
  - VCF9232: Favor/Oppose using torture on suspected terrorists
  - VCF0878: Favor/Oppose adoption by gay parents
  - VCF0879: Increase/Decrease Immigration (6 category)
  - VCF0838: By Law, When Should Abortion Be Allowed
  - VCF9238: Government should make it easier/harder to buy a gun
  - VCF9236: Does R favor or oppose the death penalty for persons convicted of murder
  - VCF9047: Federal Spending—Improve and Protect the Environment
  - VCF0890: Federal Spending—Public Schools
2. Handling of missing data:
  - Unless otherwise specified, missing data resulted in listwise deletion.
  - For 7-point party identification, DK/NAs are coded as independents at stage 1 and as pure independents at stage 2 of the branching question.

**Appendix Table B1.** Factor loading for ideology in two dimensions, 2012 and 2016.

	2012			2016		
	Uniqueness	Factor 1	Factor 2	Uniqueness	Factor 1	Factor 2
Guarantee jobs	0.502	0.692	0.143	0.595	0.425	0.474
Govt insurance	0.546	0.607	0.292	0.564	0.299	0.433
Aid to blacks	0.541	0.669		0.425	0.318	0.688
Isolationism	0.998			0.983		−0.131
Outsourcing	0.966		0.162	0.960	0.109	−0.169
Torture	0.894	−0.288	−0.154	0.793	−0.235	−0.390
Gay adoption	0.664		0.577	0.858	0.371	
Imm (how much)	0.955	0.187		0.738	0.249	0.448
Abortion	0.653		−0.589	0.818	−0.411	−0.114
Gun control	0.813	0.370	0.225	0.747	0.438	0.248
Death penalty	0.865	−0.365		0.761	−0.143	−0.467
Enviro $	0.636	0.482	0.362	0.507	0.686	0.148
Education $	0.709	0.404	0.357	0.689	0.557	
SS loadings		2.092	1.167		1.959	1.603
Proportion var		0.161	0.090		0.151	0.123
Cumulative var		0.161	0.251		0.151	0.274
Chi-squared		1584.79			1489.20	

## Appendix C. Additional Validity Checks

**1. Additional Validity Checks from 2012 and 2016 ANES**

**Appendix Table C1.** Correlations between 7-point PID and party feeling thermometers, 2012 and 2016 American National Election Studies.

	2012		2016	
Correlation between 7-point party identification and…	Live-interviewer	Self-administered	Live-interviewer	Self-administered
Dem feeling thermometer	−0.741	−0.755	−0.725	−0.752
Rep feeling thermometer	0.647	0.715	0.601	0.658

*Note*: Data are from the 2012 and 2016 ANES. Cell entries are Pearson’s correlation coefficients.

**2. Additional Validity Checks from 2019 YouGov Survey**

**Appendix Table C2.** How did independents vote in 2016 based on experimental conditions?

	Form A (Neither and not sure)			Form B (Not sure only)			Form C (Skips only)		
Voted for…	Leans Dem (%)	Pure Ind (%)	Lean Rep (%)	Leans Dem (%)	Pure Ind (%)	Lean Rep (%)	Leans Dem (%)	Pure Ind (%)	Lean Rep (%)
Clinton	57.4	23.0	7.3	54.1	18.8	4.4	49.4	0.0	5.3
Trump	4.9	26.0	80.5	4.9	30.4	79.4	8.1	33.3	64.0
Other	14.8	19.0	7.3	18.0	15.9	7.4	12.6	66.7	8.8
Did not vote	23.0	32.0	4.9	23.0	34.8	8.8	29.9	0.0	21.9

*Note*: Cell entries are percentages from a cross-tabulation of Independent party ID, split by classification as leaning Democrat, pure Independent, and leaning Republican, given the 3 experimental conditions outlined in table 2. By form, the classification of pure independents are as follows: Form A (49.3 percent), Form B (34.8 percent), Form C (1.5 percent).
